# The central role of serotonin

**DOI:** 10.7554/eLife.42291

**Published:** 2018-10-23

**Authors:** Gary C Mouradian, Matthew R Hodges

**Affiliations:** 1 Department of PhysiologyMedical College of WisconsinMilwaukeeUnited States; 2 Neuroscience Research CenterMedical College of WisconsinMilwaukeeUnited States

**Keywords:** autoresuscitation, neonatal, raphe, serotonergic system, chemogenetics, SIDS, Mouse

## Abstract

The neurotransmitter serotonin helps to co-ordinate the respiratory and cardiovascular responses of newborns to oxygen deprivation.

**Related research article** Dosumu-Johnson RT, Cocoran AE, Chang Y, Nattie E, Dymecki AM. 2018. Acute perturbation of Pet1-neuron activity in neonatal mice impairs cardiorespiratory homeostatic recovery *eLife* **7**:e37857. doi: 10.7554/eLife.37857

Immediately after birth, human infants transition from an environment in which they do not have to breathe to one in which they do. Simultaneous with this transition are major changes in blood circulation that require the cardiovascular and respiratory systems – which are not fully mature at birth – to work together, under the control of the developing brain. However, the failure of these systems, and the failure of the autoresuscitation reflex in particular, is thought to be a major contributor to sudden infant death syndrome (SIDS).

Mortality rates for SIDS steadily declined as a result of a major education campaign in the early 1990s, the Back to Sleep campaign, which recommended to parents that they place infants on their backs rather than their stomachs to sleep. However, SIDS is still a leading cause of post-neonatal mortality, and the fact that nearly 90% of cases occur between two and four months of age suggests that SIDS might result from some form of neurobiological dysfunction during this period (Filiano and Kinney, 1994). Although physiological data from infants who later succumb to SIDS are rare, it is known that SIDS is often preceded by the cessation of breathing (apnea) and/or an associated slowing of the heart rate, impaired autoresuscitation, and a failure to arouse from sleep (Meny et al., 1994; Thach, 2015). Normally, these events lead to reduced levels of oxygen and increased levels of carbon dioxide in the blood, which elicits the autoresuscitation reflex in the form of gasping for air and the restoration of a normal heart rate and breathing pattern.

A breakthrough in the field resulted from a series of careful analyses of brainstem tissues from human SIDS cases, which revealed a number of alterations in the serotonin system in the brainstem (Paterson et al., 2006; Duncan et al., 2010). These results led to the development of animal models that allowed researchers to establish links between deficits in serotonin and poor autoresuscitation. Now, in eLife, Susan Dymecki, Eugene Nattie and colleagues at Harvard Medical School and the Geisel School of Medicine at Dartmouth – including Ryan Dosumu-Johnson as first author – report the results of experiments on a new mouse model that shed new light on the role of serotonin neurons in the autoresuscitory reflex (Dosumu-Johnson et al., 2018).

The researchers show that successful autoresuscitation after repeated bouts of anoxia (oxygen deprivation) requires the serotonin neurons to be working normally within a matter of a few days after birth. When a chemogenetic approach was used to silence the serotonin neurons, Dosumu-Johnson et al. found that aspects of gasping after repeated apnea were significantly altered, whereas the effect on the heart rate was less pronounced. The failure of autoresuscitation in this model lead to substantial mortality, consistent with prior reports (Cummings et al., 2009; Cummings et al., 2011; Barrett et al., 2016). The results are also strong evidence that the respiratory reflex (i.e., gasping) and the cardiovascular reflex (i.e., increased heart rate) become uncoupled when the serotonin neurons are silenced. It has long been thought that this sort of uncoupling contributes to SIDS, but this had not been demonstrated before.

Even more novel are the findings that the gasps elicited by anoxia are not normal. First, acute serotonin neuron silencing delayed the start of the gasping reflex, which suggests that the activity of these neurons is integral to the rapid initiation of this reflex. Second, the normal linear relationship between the recovery of a robust heart rate and the recovery of a normal breathing pattern was disrupted as early as the first gasps in the serotonin-neuron-silenced mice. Moreover, many features of the first attempts at gasping were predictive as to whether or not an individual pup would go on to survive the challenges. Measurements of the ratio of ventilation relative to metabolic rate were also predictive of future failure to recover from anoxia. The demonstration of altered gasp features also counters the view that while serotonin is important for the transition from normal breathing patterns to gasping, it is not required for normal gasp-related breathing patterns (Leiter, 2009).

This work of Dosumu-Johnson et al. represents a significant advance in defining a plausible connection between serotonin system dysfunction and the vital homeostatic reflexes that are thought to fail in human SIDS. This work goes even further in defining cardiorespiratory features that may, eventually, help with the development of prevention strategies and screening tools to determine relative risk for SIDS in human infants.
